# Editorial Comment: Translational research in urology: nutricion and prostate

**DOI:** 10.1590/S1677-5538.IBJU.2020.1114.1

**Published:** 2021-07-23

**Authors:** Luciano A. Favorito

**Affiliations:** 1 Universidade do Estado do Rio de Janeiro Unidade de Pesquisa Urogenital Rio de JaneiroRJ Brasil Unidade de Pesquisa Urogenital - Universidade do Estado do Rio de Janeiro - Uerj, Rio de Janeiro, RJ, Brasil

## COMMENT

In this paper of the Urogenital Research Unit from Brazil we observe the importance of translational research in urology (1). Basic research in experimental models, human kidneys and human fetuses brought important information that helped in the understanding of various pathologies and surgeries in urology (2–4).

The authors comment in this paper that early weaning can predispose the offspring to greater risk of developing chronic diseases in adulthood and that the consumption of functional foods is able to prevent these effects. The authors performed a experimental study in rats and shows that early weaning resulted in hyperglycemia and important morphological changes in the prostate. During the experimentation the authors shows that dietary supplementation with cocoa powder attenuated these effects on the metabolism and prostatic histoarchitecture, proving to be a good nutritional treatment strategy.
